# A quasi-experimental cross-disciplinary evaluation of the impacts of education outside the classroom on pupils’ physical activity, well-being and learning: the TEACHOUT study protocol

**DOI:** 10.1186/s12889-016-3780-8

**Published:** 2016-10-24

**Authors:** Glen Nielsen, Erik Mygind, Mads Bølling, Camilla Roed Otte, Mikkel Bo Schneller, Jasper Schipperijn, Niels Ejbye-Ernst, Peter Bentsen

**Affiliations:** 1 Department of Nutrition, Exercise and Sports, University of Copenhagen, Copenhagen, Denmark; 2 Department of Geosciences and Natural Resource Management, University of Copenhagen, Copenhagen, Denmark; 3 Health Promotion Research, Steno Diabetes Center, Gentofte, Denmark; 4 Department of Clinical Biomechanics and Sport Science, University of Southern Denmark, Odense, Denmark; 5 VIA University College, Aarhus, Denmark

**Keywords:** Study protocol, Controlled study, Physical activity, Study design, Mixed methods, School-based health promotion, *udeskole*, Mental health, Learning, Education outside the classroom

## Abstract

**Background:**

Education Outside the Classroom (EOTC) is a teaching method that aims to promote schoolchildren’s learning, physical activity (PA), social relations, motivation, and well-being. EOTC activities are characterized by teachers using the local environment in their teaching, and involve innovative teaching methods, child-led approaches to problem-solving, experimentation, cooperation, PA, and play. EOTC has become common practice for many teachers in Scandinavia; however, only case studies have evaluated its impacts.

The TEACHOUT study aims to evaluate the impacts of EOTC on Danish schoolchildren’s PA, social relations, motivation, well-being, and learning.

**Methods:**

TEACHOUT is a quasi-experimental, cross-disciplinary study. Sixteen schools participated, containing 19 EOTC school classes and 19 parallel non-EOTC classes, with a total of 834 children aged 9 to 13 years. Measures of the children’s social relations, motivation for school, well-being, and academic performance were collected at the beginning and end of the school year. Data on PA levels were collected over ten-day periods during the school year using accelerometers. The amount and characteristics of the actual EOTC provided in both EOTC and non-EOTC classes were monitored day-to-day throughout the school year, using an online teacher survey platform. The effects of EOTC are mainly analysed by comparing EOTC pupils to non-EOTC (i.e. control) pupils based on their scores on the outcome variables (i.e. school performance, well-being, motivation, and social relations) at the end of the school year, adjusting for the baseline values (from the beginning of the year). The impacts of EOTC on PA are evaluated by comparing the total as well as context-specific amounts of PA of children participating in EOTC to those of children in their parallel non-EOTC classes. Furthermore, the interdependencies between PA, social relations, well-being, motivation, and learning are explored using path analysis. To help describe and understand the processes that have led to the quantitative outcomes, qualitative case observations of children’s practices and interactions in EOTC as well as classroom teaching were carried out and combined with qualitative interviews about children’s perceptions of these practices.

**Discussion:**

The TEACHOUT study represents a holistic multidisciplinary approach to educational and school health-promotion research through its study design and combination of scientific disciplines and methods, as well as its focus on the interdependent relations between learning, PA, social relations, well-being, and motivation. This will result in a comprehensive picture of school health promotion and children’s health and well-being, which will broaden the understanding of the potential benefits of EOTC in school health promotion and primary education. These results can be used to inform and guide future policy and practice.

## Background

The World Health Organization, as well as many national health organizations, regards schools as an important setting for a wide range of health-promotion initiatives [1, 2], as children spend around 40 % of their waking hours at school and as children from all socio-economic and cultural backgrounds can be reached [3]. School-based health-promotion initiatives cover almost every conceivable health topic, e.g. obesity [4–6], physical activity and fitness [7–10], fruit and vegetable intake [11], and mental health, well-being and emotional competences [12–14]. A recent study showed that in a random sample of 69 Danish schools, 61 % participated in more than three different health-promoting activities in 2010 [15].

However, school-based health-promotion initiatives are often extracurricular activities or “add-ons” to schools’ and teachers’ main objectives and everyday practice [16]. The fact that it is an extra task on top of other teaching obligations can act as a barrier to the implementation of school-based health-promotion initiatives [16], and this may be one of the reasons why the results of some school-based health-promotion interventions are mixed [17]. Integrating evidence-based health promotion with schools’ main aims and objectives in such a way that teachers and students experience them as “add-ins” rather than add-ons may help the implementation.

Education Outside the Classroom (EOTC), also called Learning or Teaching Outside the Classroom, [18] is an example of such an add-in, or holistic school-based health-promotion strategy, as it aims to promote learning, PA, social relations, motivation, and well-being [19]. EOTC activities are characterized by teachers using the local environment when teaching specific subjects and curriculum areas; for example, measuring and calculating the volume of trees in mathematics, writing poems in and about nature for language-related tasks, or visiting historically significant places in history education [18]. EOTC often involves innovative teaching methods, child-led approaches to problem-solving, experimentation, cooperation, PA, and play [19–21]. It is an educational approach characterized by action-centred and thematic learning processes involving outdoor activities [22], with the aim of promoting learning through practical observation and the use of one’s body and senses in authentic situations, and through the interaction between one’s actions and thoughts [22, 23].

In Denmark and other Scandinavian countries, EOTC is often practised through a concept called *udeskole* [20, 22]. In Scandinavia, this practice has increased markedly in the past decade. In Denmark, for example, from just a few teachers using the approach at the turn of the century, by 2007 more than 290 schools (appx. 14 % of all schools in the country) were using it [24]. Until recently, EOTC has been initiated through local development projects by individual teachers, groups of teachers, or whole schools. It has been a form of counterculture to existing education and teaching approaches, with local schools and teachers initiating it on a regular basis, e.g. one day weekly or fortnightly [20, 24]. EOTC is not a statutory requirement in the Danish school system, so the decision to take the teaching outdoors rests with the individual teacher and school. In 2014, a new national school reform was initiated in Denmark focusing on academic standards, well-being, PA, and new and more varied forms of teaching [25]. This has led to EOTC now being recommended and endorsed on a national level as one of the methods of achieving these goals [25]. Despite the widespread provision of EOTC in Denmark and the national policy recommendations, no formal systematic, structured evaluation of EOTC has been carried out. It is the aim of the TEACHOUT study to fill this gap. However, smaller case studies have been conducted. These have shown that, in the Scandinavian context, EOTC can have a positive influence on schoolchildren’s PA [26, 27], use of language [28], social relations [29], well-being [23], and attitudes to school [30].

The TEACHOUT study is a large-scale, quasi-experimental cross-disciplinary study and evaluation of the impacts of EOTC on Danish schoolchildren’s PA, learning, social relations, and well-being. EOTC is a complex intervention, requiring a detailed description of the study protocol and evaluation methods [31]. Presenting and discussing the study protocol is relevant for other research [32] on education and schools as a setting for health promotion that focuses on the interdependencies between PA, social relations, well-being, and learning. Therefore, this paper will present and discuss the study design as well as the measurements and analytical strategies used in the TEACHOUT study.

## Design & Methods

### Setting: the Danish school system

Primary and lower-secondary schools in Denmark have the same general curricular structure in all parts of the country. Schools typically have classes ranging from 0 to 10^th^ grades (6–17 years old) divided into three sections: junior (0–3^rd^ grades, 6–10 years old), middle (4^th^–6^th^ grades, 10–13 years old) and senior (7^th^–10^th^ grades, 13–17 years old). Each class contains a maximum of 28 gender-mixed pupils [33, 34].

The Danish Parliament legislates the overall aims of education in both private and public schools, and the Minister of Education establishes targets for each subject. However, the local municipalities, schools and teachers decide how to achieve these targets; they have so-called “freedom of methods” [33, 35].

### Study aims and study design

#### Research aim

**Fig. 1:**
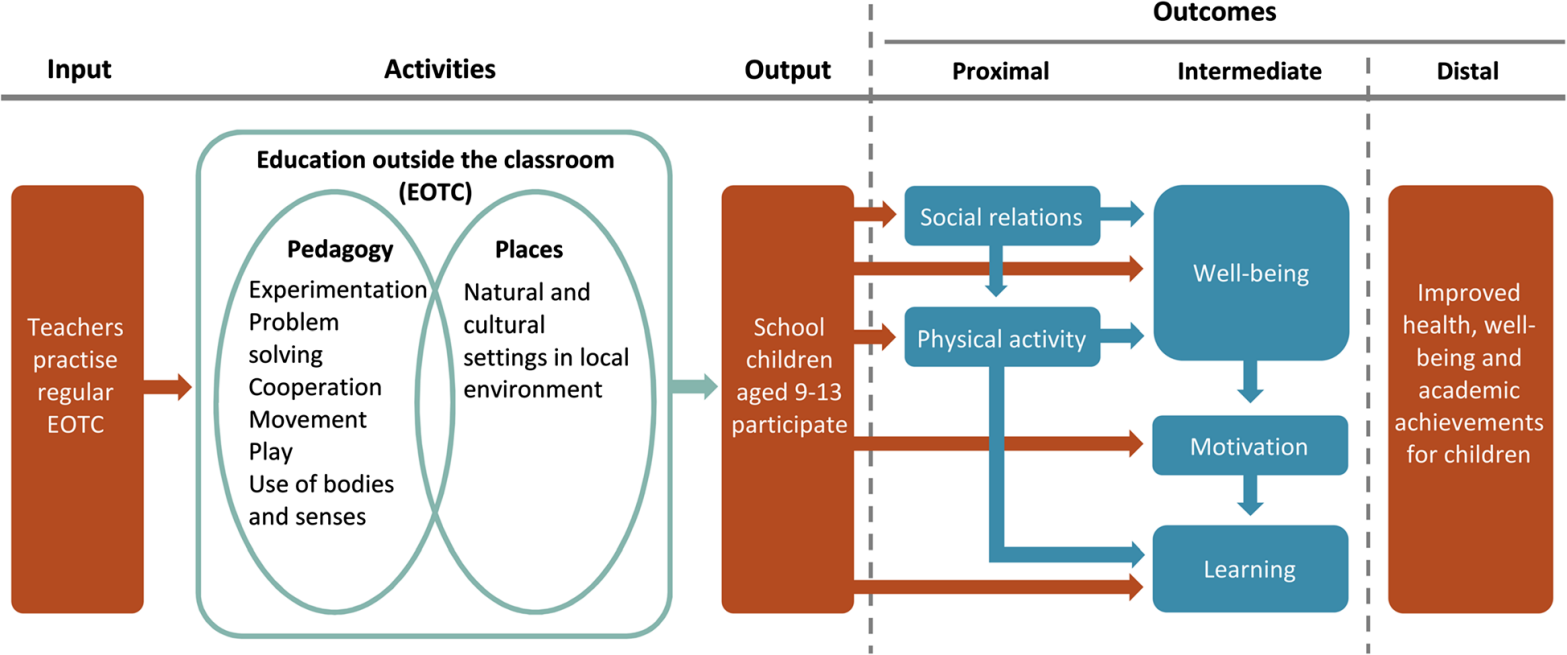
Programme theory of potential effects of EOTC and its various programme elements and potential outcomes

The objective of the TEACHOUT study is to understand how regular EOTC influences PA, learning, social relations, motivation, and well-being among schoolchildren in the 3^rd^ to 6^th^ grades (9–13 years of age). The programme theory of the potential effects of EOTC and its various programme elements and potential outcomes is illustrated in Fig. 1. The main research question is: *Does EOTC increase and improve schoolchildren’s physical activity, academic learning, social relations, well-being, and motivation at school? If so, how?*

This question will be investigated for all schoolchildren, as well as for target groups for whom EOTC is thought to have special benefits. The latter include children who are overweight or physically inactive, or have special needs and difficulties in terms of social interaction or learning at school. The study will provide knowledge and broaden our understanding of the potential benefits of EOTC, and reveal whether and how EOTC is a relevant complement to both health promotion and the promotion of children’s well-being and learning in schools.

#### Study design

The study builds on a quasi-experimental design, is cross-disciplinary, and uses mixed-methods evaluation. It is quasi-experimental in the sense that it compares the developments in school performance, social relations, well-being, and motivation as well as the differences in daily PA among children in school classes which by their teachers’ choice (i.e. self-selection) use EOTC as a teaching method to those of children in their parallel classes which do not use this method. The study is cross-disciplinary as it examines the interrelations between different phenomena such as teaching methods, teaching context, social relations, learning, and PA, which are subjects belonging to and studied in different disciplines such as pedagogy; social, cognitive and developmental psychology; sociology; and public health. The investigators of the study have various scientific backgrounds: sociology, psychology, exercise physiology, geography, public health, and education.

**Fig. 2:**
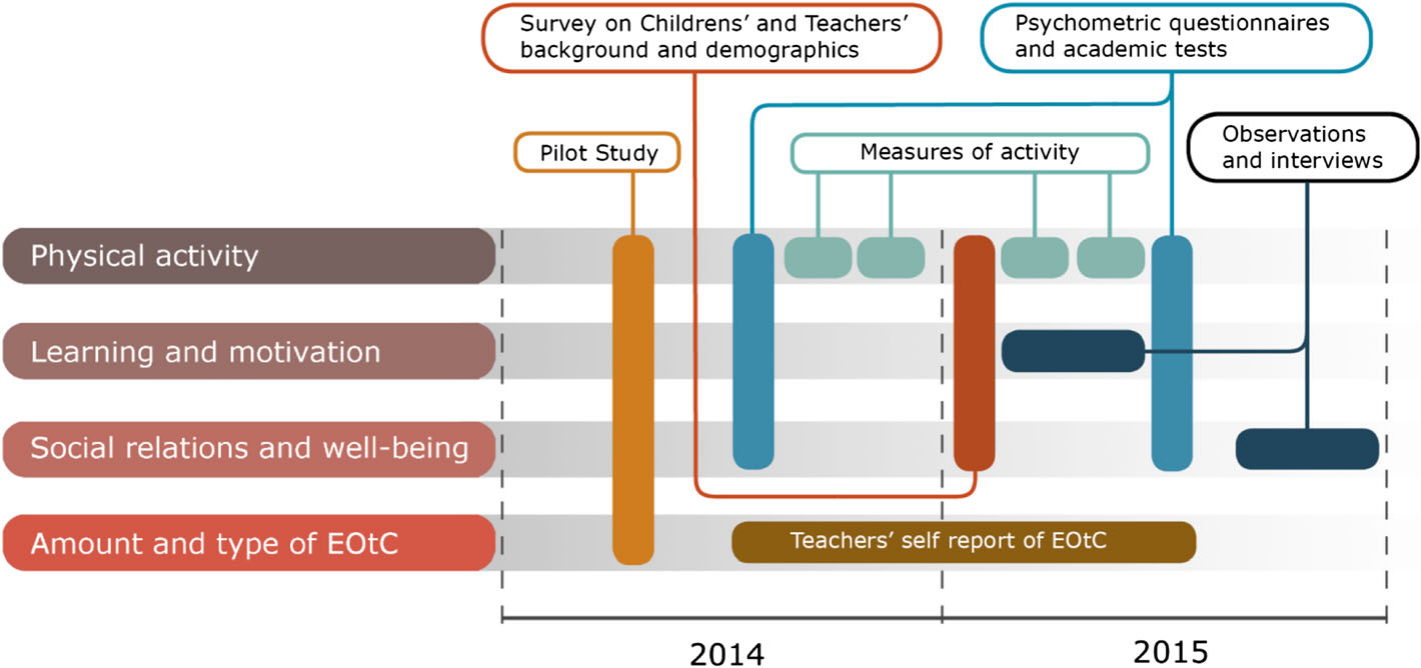
Main parts and overall timeline of the study

Finally, the study is mixed-methods, as a range of qualitative and quantitative methods are employed and combined. The study design, with its different sub-studies and phases, is illustrated in Fig. 2.

### Sampling and participants

Conceptually, EOTC is defined as regularly held education outside the classroom in natural and cultural settings [20, 22]. In order to operationalize this concept into measurable criteria, we defined EOTC school classes as school classes that practise teaching outside the classroom and school buildings for an average of at least five hours a week over the school year. The minimum of five hours of EOTC per week was chosen to indicate that classes were exposed to EOTC approximately one day per week, and hence had EOTC as a substantial and regular part of their school life (as opposed to, e.g., occasional field trips). In order to have both a group of schoolchildren participating in EOTC and a group not participating who were otherwise similar and comparable, school classes were recruited in pairs such that each EOTC class had a non-EOTC comparison/control class at the same school and grade level. In the Danish school system pupils in each school are randomly assigned to a class, which means that pupils in parallel classes are comparable in background characteristics and in environmental surroundings at school and in the local community.

Therefore, the inclusion criteria for schools were that they agreed to have at least one class (in grades 3–6) which would practise EOTC for an average of at least five hours a week during the school year Aug 2014–June 2015, and at least one parallel class at the same grade level that would not use EOTC and was willing to participate as a control.

Because the distribution of pupils into classes within schools and grade levels is random [33], the use of parallel non-EOTC classes as a control group ensured that these two groups had comparable parental backgrounds, local areas and overall school resources. However, these criteria also excluded the many schools that use EOTC for all classes on the same grade level from participating in the study. We recruited schools in different regions of the country as well as those in rural, suburban and urban areas.

Schools were contacted and recruited using three different approaches. In a national survey of Danish schools [24], 290 of the responding schools answered that they used EOTC. As we already had their contact details, these 290 schools were contacted directly. Furthermore, we contacted the municipalities in the five different regions of Denmark to gather information on which of their schools were using EOTC, and to obtain permission to contact these schools. Finally, we used our personal and professional networks to contact potential schools and teachers. Ultimately, of 1,313 Danish schools, 549 were contacted to determine whether they met the inclusion criteria and were interested in participating in the study.

Sixteen schools with a total of 19 EOTC classes and 19 parallel non-EOTC classes met the inclusion criteria and agreed to participate in the study, entailing baseline measurements in August 2014 and end-line measurements in May 2015. However, as we did not receive consent forms from the children at one school (with one EOTC and control class) at baseline measure, only 18 EOTC and 18 control classes were included in the measurements of children’s development from the start to the end of the school year. In total, 769 children from 18 EOTC and 18 control classes provided consent and had their pre and post measures included in the data sets for effect analysis.

**Fig. 3:**
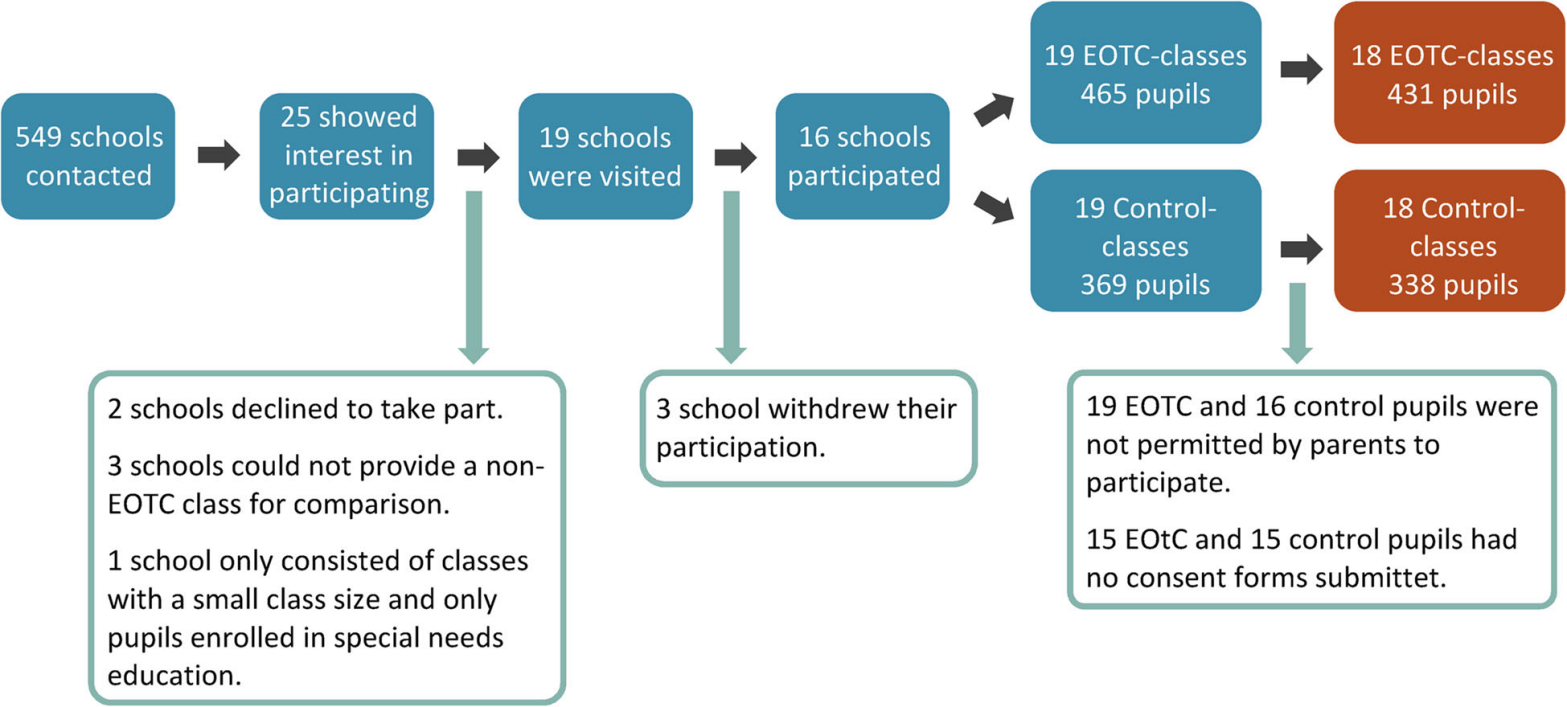
Flowchart of recruitment stages, number of excluded and included schools, and reasons for inclusion/exclusion as well as numbers of participating pupils

A flowchart mapping the various stages of recruitment, number of excluded and included schools, and reasons for inclusion/exclusion as well as the numbers of participating pupils can be seen in Fig. 3.

The large discrepancy between the number of schools contacted and those participating is due to multiple reasons. Many of the schools were not interested in or willing to use EOTC under our defined criteria; others were not able to meet the inclusion criterion of a non-EOTC comparison class; and finally, some did not wish to participate due to lack of time. The final consent for a school’s participation was given by its principal. Each individual teacher involved in teaching EOTC and/or data collection also gave consent participate.

**Fig. 4:**
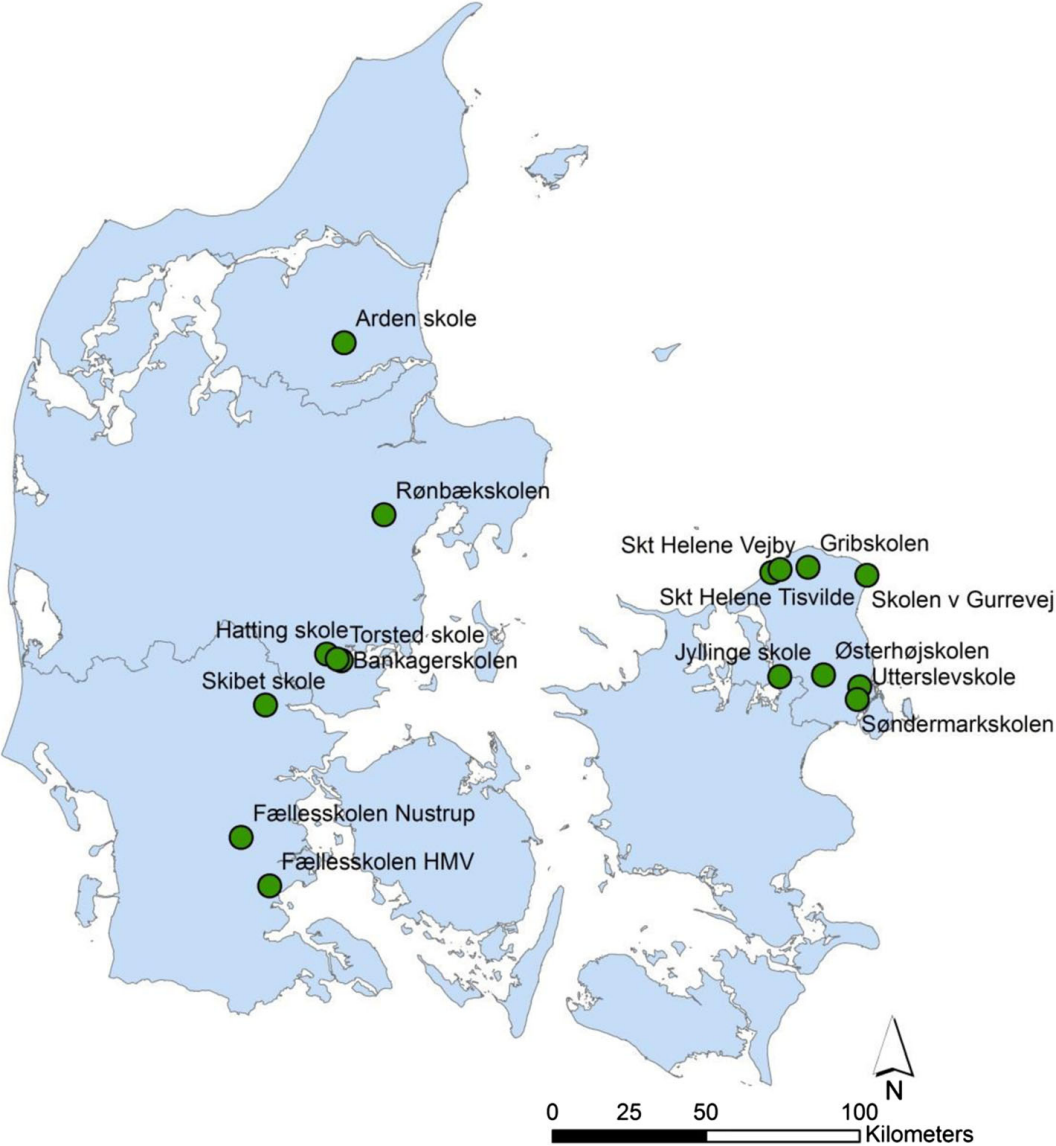
Map of Denmark with locations of participating schools

The participating schools are distributed in the different regions of Denmark (see Fig. 4) but with the largest concentration in the region around the capital, Copenhagen, and the eastern part of Jutland. As can be seen in Table 1, the participating schools are located in rural as well as suburban and urban areas of varying sizes, economic backgrounds, housing densities, and resources for EOTC, in terms of nearby access to green spaces and nature.

**Table 1:** Description of the 16 participating schools in terms of their size and the geographic and economic resources of their nearby surroundings

	Range	Mean (SD)	Danish average
Number of pupils	117–2002	665 (449)	299
Average household income in DKR	561440–834359	678773 (74723)	666246
Number of households within 10 km^a^	6567–609486	108651 (197381)	46430
Distance (meters) to the nearest green space	45–962	381 (253)	320
Square meters of green space within 10km^a^	36447–17339806	3048046 (5655208)	2856900
Number of green spaces within 10km^a^	3–53	23 (13)	12

Green space was defined as all parks, woodlands, nature areas and heathlands registered in the official land use database of The Danish Geo data Agency. ^a^of the school

### Support and encouragement for participating teachers

For the success of the study, we found it important to meet the EOTC teachers face to face in order to inform them about the inclusion criteria, to explain how we as researchers had decided to define EOTC, and to answer their questions. Therefore, the EOTC teachers in the study were invited to a two-day seminar where workshops and networking took place and more in-depth information about the study was provided. These teachers were an important link for the involvement of their colleagues in the parallel non-EOTC classes. We also met with the non-EOTC teachers during visits to the schools, to give them information and answer their questions.

In order to show our appreciation for the teachers’ efforts in organizing questionnaires, collecting accelerometers, contacting parents and registering their EOTC activities weekly, a gift certificate of 500 DKK (67 EUR) was given twice to all teachers for their personal use, midway and at the end of the study.

### Measurements and data collection

All data were collected between August 2014 and June 2015. Both longitudinal (repeated measures) and cross-sectional data were collected.

#### Repeated measures

In order to assess the effects of EOTC on children’s social relations, motivation for school, well-being, and school performance, measures of these constructs were collected at the start of the school year (August 2014) as well as the end (May 2015) for both the children in the EOTC classes and those in their parallel non-EOTC classes. In order to minimize the response burden per questionnaire and test, these measures were collected on different occasions. School performance tests in reading and mathematics were both done on paper during class hours on separate occasions. Measures of well-being and motivation, as well as the learning rating scale, were collected through an electronic questionnaire during class on a separate occasion. This questionnaire took an average of 20 min for the children to complete. Measures of the children’s social relations were collected on a paper-form questionnaire during class on a separate occasion; this questionnaire took an average of ten minutes for the children to complete.

#### Cross-sectional data

In order to measure the PA levels in different contexts, such as days with and days without EOTC, and in order to compare the total amounts of PA of the children in EOTC classes to those in non-EOTC classes, accelerometer measures of PA combined with school timetables and activity questionnaires were collected for one ten-day period per school, with an EOTC class and its parallel non-EOTC class measured on the same days. Data from different schools were collected throughout the school year.

Data on the children’s backgrounds and their teachers’ qualifications were collected through an online survey, conducted with both the children’s parents and their teachers.

In order to measure the extent and types of EOTC to which all the children in the study were exposed during the year, teachers were asked to report information on this every week of the school year in an online questionnaire. Table 2 depicts the different measures and the time of year they were collected. All the measures are described in more detail below.

**Table 2:** The different measures collected and the time of year they were collected

Construct/Measure	Instrument/method for data collection	Number of items/tasks	Time of collection
Pupils’ academic performance in Reading and Mathematics	Sentence reading test [36] Mathematical basic skills test [37]	Reading: 15–24 tasks Maths: 50–87 tasks	August 2014 and May 2015
Pupils’ social relations	Social Network Analysis Social Cognitive Mapping	21	August 2014 and May 2015
Pupils’ well-being	Strength and Difficulty Questionnaire [44]	25	August 2014 and May 2015
Pupils’ motivation for school	Academic Self-Regulation Questionnaire [51]	17	August 2014 and May 2015
Pupils’ physical activity	Axivity, AX3 accelerometers		10 day periods from November 2014 to June 2015
Contexts of the PA	Schools’ class time tables Activity questionnaires	Questionnaire: 50 items	10 day periods from November 2014 to June 2015
Pupils’ height, weight and BMI	Height Measure Body Composition Monitor		At the beginning of the 10 day periods from November 2014 to June 2015
Pupils’ background	Electronic Questionnaire to parents	33	March 2015
Teachers’ background	Electronic questionnaire	13	March 2015
Degree of implementation of EOTC	Online platform	16	Throughout the school year
Processes and interactions of importance to social relations, well-being and motivation	Qualitative case observations, focus-group interviews and personal interviews		Throughout the school year
Learning processes	Qualitative case observations, focus-group interviews and personal interviews		Throughout the school year

### Measures

#### Pupils’ academic performance

The children’s reading comprehension in Danish was measured using the validated age-adapted sentence reading test (*Sætningslæseprøve*) by Hogrefe [36]. Test levels 3–6 (i.e. 3^rd^ to 6^th^ grades, 9 to 13 years of age) were used. For each grade level, the same test was used at the beginning and end of the school year. The test was taken on paper during class as instructed by the teacher who taught Danish, who was familiar with it. The test has been validated, and assesses reading ability as the combination of speed and level of comprehension (measured as the percentage of correct answers regarding the meaning of texts per minute used to complete the test) [36]. The number of correct answers per minute was used to test the effects of EOTC on pupils’ abilities to read and understand.

The children’s abilities in mathematics were measured using the validated age-adapted mathematical basic skills (MG) test by Hogrefe [37]. The test was taken on paper during class as instructed by the teacher who taught mathematics, who was familiar with it. The MG test measures skills within the main subjects/themes of mathematics, defined by the age-specific curriculum (e.g. geometry, calculus, diagrams, etc.). With increasing grade levels, more subjects are tested and therefore the test becomes longer. We used test levels 3–6 (3^rd^ to 6^th^ grades, 9 to 13 years of age), which consists of 50 to 87 tasks depending on level. The test measures the pupils’ levels of ability in mathematics in general as the number and percentage of correct answers in total. It also assesses the level of ability on three ordinal categorical levels of skill acquirement (not acquired, insecure, and secure) within the various mathematical subjects tested. The percentage of correct answers in total was used to assess the effects of EOTC on the children’s general skills in mathematics.

The Learning Rating Scale [38] was used to measure the degree to which learning processes were facilitated in the classroom. Using five questions answered on a 1–10 VAS scale, this scale assesses the pupils’ perceptions of how much they learn at school, how well they are getting along, and how well the teaching methods fit them.

#### Pupils’ social relations

The strength and diversity of pupil-pupil social relations as well as class social inclusion and coherence were measured using general Social Network Analysis (SNA) approaches [39, 40] and Social Cognitive Mapping (SCM) [41, 42]. We developed questions to measure the pupils’ interaction frequency and social networks, covering both affective (i.e. friendship/“hanging out with”) and instrumental social relations (i.e. collaboration and helpfulness). Each pupil was asked to list: a) up to eight groups from their class who hang around together a lot during breaks; b) up to five peers they hang around with a lot during breaks; and c) up to five peers they sometimes help, or receive help from, with schoolwork and homework.

In addition, each pupil was asked to confidentially name up to three classmates they would describe as having one of the following nine characteristics: “Cooperative”, “Starts fights”, “Popular”, “Athletic”, “Disruptive”, “Leader”, “Good at schoolwork”, “Not so good at schoolwork”, and “Shy/withdrawn”. To minimize ethical concerns, we were careful regarding the procedures for collecting these data. In the description of each characteristic great care was taken to avoid valuing them as good or bad, by describing them as common and natural (e.g. “Many children find schoolwork difficult. Please name three classmates who sometimes struggle with their schoolwork”). The children were told by their teachers they could not reveal their answers to others. The questionnaire was completed on paper, with enough distance between the children that they could not see each other’s answers. When giving consent, parents and teachers were informed about this questionnaire, and the children were informed that they could drop out of any part of the study they wanted to. This peer assessment approach [41–43] provides information on prominent pupils in the identified group clusters, making it possible to assess the groups’ norms and values. SNA and SCM data will be combined in order to construct the social networks of each participating class community, and prominent group clusters will be determined in order to assess levels and characteristics of inclusion and interpret patterns of interaction.

#### Pupils’ well-being

The children’s well-being was measured using the validated Danish version [44] of the children’s self-report version of the Strength and Difficulty Questionnaire (SDQ) [45, 46]. This instrument is a validated, widely used questionnaire measuring five aspects of children’s difficulties and strengths in relation to their surroundings: “emotional symptoms”, “conduct problems”, “hyperactivity/inattention”, “peer relationship problems”, and “pro-social behaviour”. The questionnaire has 25 items, answered on a four-point Likert scale and added up to give scores for each of the five measured strengths and difficulties. The advantage of using the SDQ is that it is commonly applied in school contexts and includes social aspects of well-being [47]. In addition, it is used in the only other study (known to us) on the well-being effects of an outdoor teaching and learning intervention [23], making comparisons possible.

#### Pupils’ motivation for school

The children’s extrinsic and intrinsic motivation for school was measured with the Academic Self-Regulation Questionnaire (called SRQ-A) [48]. The SRQ-A is a domain-specific self-report questionnaire developed for measuring the level of autonomy relative to doing different types of schoolwork among pupils in late primary and lower-secondary school. The SRQ-A is based on Self-Determination Theory [49, 50] and is developed for and validity tested on children in elementary school. The questionnaire consists of 17 items that measure motivation for schoolwork through one general measure of intrinsic motivation, and three sub-scales of extrinsic motivation (External Regulation, Introjected Regulation, and Identified Regulation). However, the SRQ-A does not measure the specific sub-scales or forms of intrinsic motivation, which may be important for understanding developments in intrinsic motivation that EOTC might support. Therefore, the SRQ-A was supplemented with the intrinsic motivation sub-scales “to know”, “to accomplish” and “to experience stimulation”, taken from the Danish version of the Sport Motivation Scale [51], which we adapted to the school context.

#### Pupils’ physical activity

PA behaviour was measured over ten consecutive days in order to assess the differences between school days with and without EOTC, as well as to adjust the analyses for PA in other settings, e.g. during PE, recess and leisure time as well as on weekend days. In order to increase compliance and reduce insecurity about wear time [52, 53], Axivity AX3 accelerometers were used to measure PA because of their small size and the possibility to fix them directly to the skin with a plaster. In order to measure different kinds of bodily movement, two Axivity AX3 accelerometers were fixed directly to the skin with tape. The first accelerometer was fixed to the right side of the child’s back just above the upper point of the posterior iliac crest, with the positive x-axis pointing downwards. The second was fixed to the right medial front of the thigh midway between the hip and knee joints, with the positive x-axis pointing downwards. If an accelerometer fell off before the end of the ten days it was not put back on (this approach was preferred in order to eliminate wear-time validation issues, even though it meant that some days of measurements could be lost).

#### Pupils’ height, weight and BMI

Height and weight were measured using the Leicester Height Measure and the OMRON BF212 Body Composition Monitor. At measurement, the children wore light clothing and no shoes. BMI was calculated, and BMI status (thinness, overweight and obesity) was calculated using age and gender-specific references [54].

#### Contexts of the pupils’ PA

In order to to identify the physical activity levels in different everyday life contexts, accelerometer data was combined with data on the times of various activities and contexts during the days of accelerometry.

Information about the setting of the measured PA was obtained through a combination of the schools’ class timetables and a diary/activity questionnaire filled in by the pupils and their parents [13, 27]. Class timetables were collected from all participating classes to allow the activity levels of different school settings, such as classroom teaching, EOTC school breaks, and PE lessons, to be quantified. In addition, in each class the teacher selected three pupils to fill in a school protocol, in which they reported whether anything during school hours turned out differently from the class timetable, e.g. if and when they had had EOTC, if recess had started earlier or later, or if they had had a substitute teacher.

To measure the times of leisure-time activities each pupil was asked to complete a standardized questionnaire-based activity diary, in cooperation with his/her parents. This covered specific times of daily activities such as waking up, going to bed, time spent at day-care institutions, active transportation lasting more than five minutes, and sports and exercise lasting more than five minutes. Pupils were also asked to report whether they had been absent from school and, if an accelerometer had fallen off, why and when this had happened.

#### School absence due to illness

The number of days in the whole school year of school absence due to illness (an outcome health variable) was registered using school protocols.

#### Pupils’ background

A short survey was conducted (May 2015) to assess the children’s socio-cultural background and personal traits of importance to their engagement in EOTC, and hence its outcome. Parents were asked about their education and occupation (in order to assess socio-economic position), the child’s and their own birthplace (in order to assess ethnic background), and the child’s participation (type and amount) in leisure-time sports as an indicator of sports capital [55]. Parents were also to report how often their child visited different types of green areas, to allow us to assess their level of familiarity with such places. Parents were also asked about any psychological or physiological disabilities their child might have (e.g. Tourette syndrome, ADHD, etc.).

#### Teachers’ background

An electronic questionnaire was developed to assess the teachers’ qualifications for EOTC teaching as well as teaching in general. All participating teachers of both EOTC and their parallel non-EOTC classes completed the questionnaire. The questionnaire asked about the teachers’ educational level, which subjects they were qualified to teach, what year they had qualified as teachers, the number of years they had been teaching, whether they had any experience in EOTC, and whether they used the outdoors as an educational location (and, if so, for which subjects).

### Exposure to EOTC during the school year

As described, the intervention (i.e. exposure) group of the study consisted of school classes planning to practise EOTC at least five hours a week, while the control classes were not expected to practise any EOTC. However, controlled studies (RCT or quasi-experimental designs) in real-life settings such as in schools need to have valid data on the extent to which both the intervention and control groups were exposed to the intervention studied [31]. Researchers cannot be sure of the extent to which interventions conducted in real-life school settings by “real” teachers are implemented as intended [56, 57]. Furthermore, in such real-life settings, researchers cannot be sure that the classes functioning as a control group are not practising any of the activities that are part of the intervention (e.g. EOTC). It is therefore crucial to monitor what is happening in practice throughout the intervention period, in order to obtain high validity data in studies like this. However, we were unable to find an appropriate tool for monitoring the extent to which teachers apply a specific teaching methodology or practice which could have been adapted for our purpose. Therefore, an online platform was developed and tested for collecting (both EOTC and control) teachers’ registration of weekly EOTC in terms of type, time, amount, subjects taught, and place. This monitoring tool enabled us to monitor whether, and to what extent, the EOTC classes involved in the study adhered to practising EOTC for a minimum of five hours weekly, as intended, and how many of the control classes adhered to not practising any. Monitoring the amounts of EOTC and the places where it was practised, as well as the subjects taught, also enabled us to study the relation between yearly amounts of EOTC and the children’s learning, social relations, well-being, motivation, and health. This analytical approach is especially important if the monitoring shows large discrepancies between the intended and the actual practised amount of EOTC in the intervention and/or control classes.

### Follow-up qualitative studies

The mixed-methods approach applied in this study was sequential quantitative-qualitative [58]. To be able to describe and understand the processes that led to the quantitative outcomes, in a follow-up we carried out and analysed qualitative case observations of children’s practices and interactions in EOTC and classroom teaching. Insights from these observations were further explored using qualitative interviews with different groups of children regarding how they perceived teaching outside and within the classroom, and what differences it made for them and their class. The qualitative studies had two main focuses: one on learning processes, and one on well-being and motivation.

#### Pupils’ learning processes

In this follow-up qualitative sub-study, we conducted qualitative observations and interviews in two strategically selected EOTC and non-EOTC classes. Both were observed twice. The foci of the observations were how the setting and the content of the teaching in interaction shaped the learning processes. These observations provided us with context-specific knowledge important for the design of the interview questions. To gain thorough insight into how EOTC influences children’s learning and memory, 40 qualitative semi-structured interviews were carried out with the children in the two classes.

#### Pupils’ well-being and motivation

This follow-up qualitative sub-study focused on how academically strong and academically challenged pupils experience and interact in EOTC, and how this influences their social and academic well-being and motivation at school. Qualitative observations and interviews were conducted. Six initial observations of EOTC and classroom teaching informed the questions for the interview guide, as well as the interpretation of the interview data. Five academically strong and five academically challenged pupils with an approximately equal gender distribution were strategically sampled based on their test results in reading and mathematics (please see *Measurements and data collection* section) and interviewed. The ten interviews were carried out at the school, and were based on a semi-structured interview guide [59].

### Data analysis

#### Statistical analysis

Confirmatory Factor Analysis will be used to test the validity of the translated scales of motivation and well-being, and Cronbach’s alpha tests will be used to test their internal consistency and reliability. The validity of the teachers’ registration of weekly EOTC will be evaluated by relating the reported locations of EOTC to GPS measures of the school classes’ location during the ten days of accelerometry.

The influence of EOTC on the children’s PA will be analysed by comparing the mean daily minutes of PA (in total and during school time) over a week of the children participating in EOTC to those of the children in their parallel non-EOTC classes, and by comparing the PA levels of the children in EOTC classes on days with EOTC to days without it.

The influence of EOTC on the children’s social relations, well-being, motivation, academic performance, BMI, and school absence will be analysed by comparing EOTC pupils to non-EOTC (control) pupils in these measures at the end of the year of EOTC, adjusting for the baseline values (from the beginning of the year) using multilevel regression analysis.

In order to test the specific effects of EOTC on relevant subgroups, interaction terms will be used to test the moderating effect of personal characteristics such as gender, socio-economic and ethnic background, leisure-time sports activity, overweight status, and psychosocial challenges (such as ADHD).

Path analysis will be used to analyse mediators of the effects of EOTC, e.g. whether its effects on learning are mediated by motivation for school, or whether its effects on PA are mediated by social inclusion.

Adjustments for multiple testing will be applied as appropriate (e.g. in the analysis of effects on the children’s abilities in different maths subjects).

#### Interrelations between social relations, physical activity and learning

It is very likely that the study’s main outcome variables–social relations, physical activity, well-being, motivation, and learning–are interrelated.

**Fig. 5:**
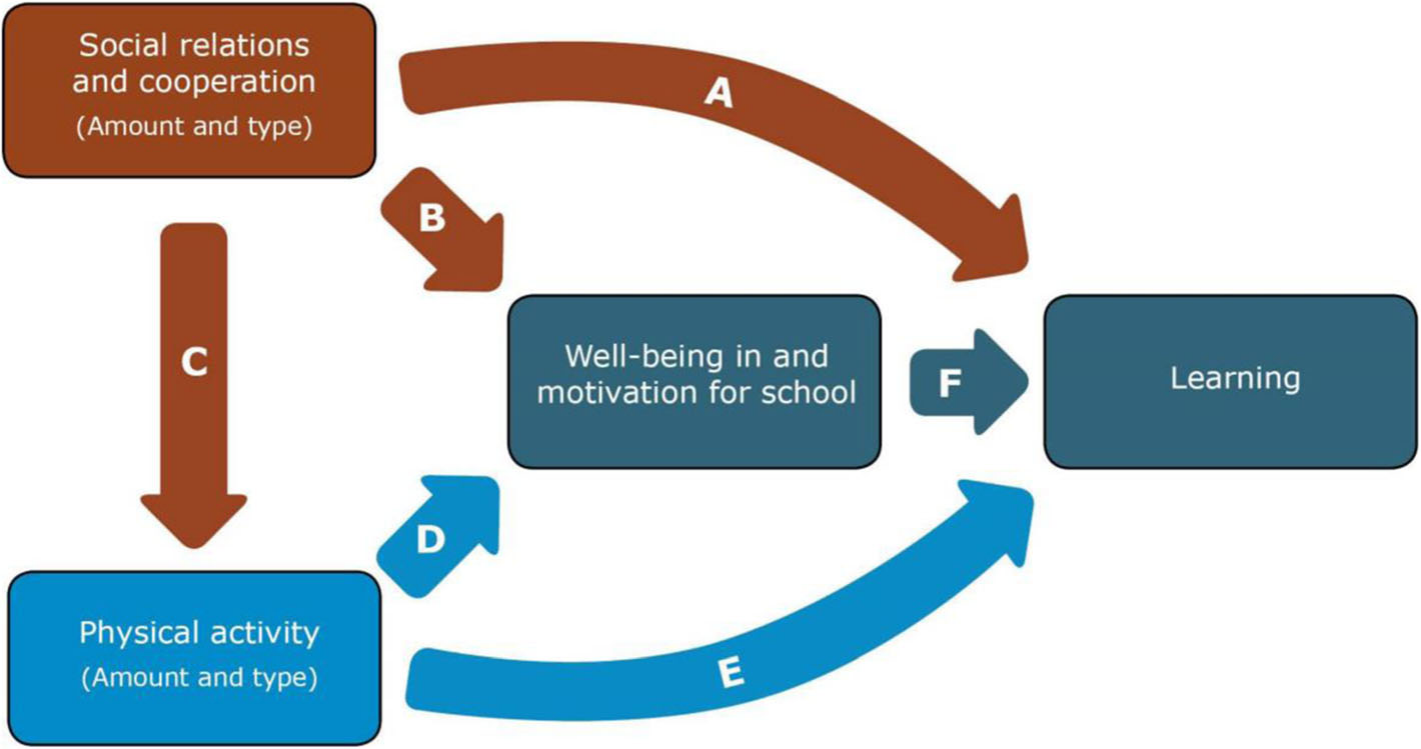
Assumed interrelations between main outcome variables. a: Deep learning through interactive negotiation, argumentation and feedback processes. b: The prevalence of positive social interaction influences how intrinsically motivating contexts and activities are. c: Inclusion in physically active games and play. d: It is difficult for children to sit still. Opportunities for movement/PA increase well-being. Some types of physical activities furthermore support feelings of positive social relations, competence and autonomy, which are central to emotional well-being. e: Three hypotheses: 1) Concrete bodily experiences of abstract/academic phenomena increase one’s understanding of them; 2) A bodily experience of what is learned helps one’s memory; 3) Physiological stimuli of neurological structures of importance to cognitive functioning. f: Involvement

Analysing this interrelatedness is important to understanding how, and hence why, EOTC and other similar teaching methods influence children’s development. One example of this interrelatedness is that physical activities among children are most often social activities in which some children are included and others are excluded [60, 61]. Therefore, social relations and social inclusion among children in school classes will have an impact on which children, and how many of them, are physically active [62–64]. Furthermore, as described in the Self Determination Theory and in research on well-being, positive social relations and inclusion are important to well-being [65] and motivation [66, 67], which in turn are important for learning [52, 68]. But the amount of PA in itself may also have an independent effect, as a physiological stimulus on cognitive functioning and hence school performance [69–71] and well-being [12, 70]. The hypothesized interrelations between social relations, PA and learning to be investigated are illustrated in Fig. 5, below. The interrelations between social relations, PA, well-being, motivation, and academic performance will be analysed using multiple regression models and path analysis, including these factors and their interaction terms.

#### Analysis of qualitative data

The observation and interview data on the pupils’ learning processes will be analysed by drawing on the revision of Bloom’s taxonomy of knowledge [72] focusing on the categories factual, conceptual, procedural, and metacognitive knowledge and processes, in order to analyse what and how the pupils have learned during their participation in either EOTC or non-EOTC. The interview data on pupils’ well-being and motivation will be analysed drawing on a qualitative hermeneutic approach including insights and understandings from the observations.

### Research ethics

By Danish law, only research projects of a biomedical character or studies involving a risk to patients/participants must have their ethics reviewed by a Regional Ethics Board; all other research projects are exempt from applying for formal ethical approval. We discussed our study with an ethics committee and received official confirmation from the Regional Committee on Research Ethics that our study did not require ethics approval, therefore, study was not assessed (protocol no.: H-4-2014-FSP). However, in Denmark all research projects that collect identifiable data are required to demonstrate that they have appropriate procedures in place to protect the data and secure participant anonymity. We reported the TEACHOUT study to the Danish Data Authority, and received formal approval of our data collection and storage procedures (ref. number: 2014-54-0638). All data have been anonymized and will only be analysed on a group level.

The teachers involved in the study were given both written and oral information about the study before they decided whether or not to participate. Parents received written information about the study, and were asked to provide written consent if they would allow their children to participate. All children received both written and oral information, and were asked to provide their assent to participate in the study, if their parents had provided written consent allowing them to participate. The children were informed that participation in the measurements was voluntary and that they could withdraw at any time.

Even though the TEACHOUT study is exempt from applying for ethical approval, there are some ethical concerns to consider. In our view, the major ones are related to the questionnaires for the social network analysis, especially the peer assessment approach. In the *Methods* section we described our attempts to minimize the ethical challenges of this method. Other studies have investigated the impacts it has on children and found that the condition of minimal risk of harm (harm not greater than children might encounter in daily life) was not breached [73]. In the TEACHOUT study, we have had no report from the teachers, parents or children concerning problems associated with the children completing the questionnaire.

## Discussion

Initially, we argued for the need and potential for integrating evidence-based health promotion with schools’ main aims and objectives (i.e. promoting learning and well-being), and presented EOTC as an example of such an “add-in”, or holistic health-promotion strategy. The aim of this paper was to present the study design, sampling, data collection, measures, and analytical strategies developed and used in the TEACHOUT study. The study investigates how regular EOTC influences pupils’ PA, learning, social relations, well-being, and motivation for school. Presenting and discussing this study protocol is relevant, because it is important to develop, implement and evaluate complex, real-life school-based health-promotion strategies that have a holistic approach and aim [74–76]. The quasi-experimental, cross-disciplinary, mixed-methods study design needed in such studies offers a number of strengths, but also limitations. The most central strengths and limitations of the TEACHOUT study are discussed below.

Even though RCTs are considered to be the gold standard for accessing the effects of an initiative or intervention [77], we deliberately chose a quasi-experimental design in which the involved EOTC school classes were not randomly assigned. The current EOTC practice in Denmark is mainly teacher-driven (i.e. it is a grassroots, bottom-up initiative [25]). It is the influence and effects of this EOTC practice that we aim to study. Therefore, randomly assigning classes to an intervention and a control group (as in a classical RCT design) was not feasible or appropriate [31]. Instead, we attempted to ensure that the exposure/intervention and control groups were comparable by using the parallel classes at the same grade level and school of each of the EOTC classes as the control group. The use of parallel non-EOTC classes as a control group ensures that the two groups are comparable in respect to confounding variables such as parental background, local area and overall school resources (as the distribution of pupils into classes within schools and school years is random in the Danish public school system). However, the EOTC and the non-EOTC groups might still be different in terms of having different teachers. As EOTC is often chosen by the teacher, it is likely that the teachers of the classes practising EOTC are different types of teachers compared to those who have not chosen to practise it. The EOTC teachers may differ regarding other factors than using EOTC as a teaching method. Examples of differences between teachers that may affect the pupils’ PA, social relations and learning are type of education, enthusiasm, pedagogical approach, and didactical values and skills. As school classes in Denmark usually have the same teachers over several years, some of these differences may already have had effects at baseline (i.e. the start of the school year) and can therefore be taken into account to some degree in statistical models when adjusting for the baseline values of the outcome being compared. However, some of the differences may affect the children during the duration of the study and may therefore introduce a selection bias on the results. We have collected data on the teachers’ educational background in order to adjust for this factor in the statistical models, but measuring other variables related to differences in the general quality of teaching should be considered in similar future studies. We do not believe that a clustered sampling with schools as a cluster would ensure the same level of comparability between the two groups. However, using parallel classes at the same schools introduces a risk of spillover effect whereby teachers using EOTC might inspire their colleagues to also use it. The possible effect of this “contamination” was taken into account by monitoring the EOTC practised by both the EOTC classes and non-EOTC control classes.

As EOTC is based on a holistic view of children’s health and development, this study also needed to be based on such an understanding. This necessitated the combination of a number of scientific disciplines and types of measures. Some argue that such a mixing of methods and scientific paradigms creates many challenges and some limitations [78], but it may also enable us answer our research question to a fuller extent [79, 80]. We believe that our cross-disciplinary approach is necessary to produce findings that are more relevant to the development of schools than can be produced by a mono-disciplinary approach alone, e.g. a classical medical health research approach, as school development and policy reform need to take into account all the possible benefits and problems associated with new health-promoting initiatives like EOTC.

## Conclusion

In conclusion, evaluating the effect of an “add-in” school health-promotion strategy through EOTC in a real-world setting is both complex and challenging. The TEACHOUT study offers a novel approach in the fields of educational and school-based health-promotion research through its study design, wide range of measures and cross-disciplinary, mixed-methods approach, as well as its “holistic” focus on learning, PA, social relations, motivation, and well-being. By merging these perspectives into one study we broaden the view on their interrelations, resulting in a comprehensive picture of school health promotion and children’s health and well-being. The study will therefore provide knowledge and broaden our understanding of the potential benefits of EOTC in school health promotion and primary education. These results can be used to inform and guide future policy and practice regarding EOTC and how to improve learning, well-being and PA for children in schools.

## Abbreviations

DKK: Danish Krones (*Danske Kroner*)
EOTC: Education outside the classroom
EUR: Euros
PA: Physical activity
SCM: Social cognitive mapping
SDQ: Strength and difficulty questionnaire
SDT: Self determination theory
SNA: Social network analysis
SRQ-A: Academic self-regulation questionnaire
